# Ligation of patent ductus venosus in a child with pulmonary arterial hypertension and hypersplenism: A case report

**DOI:** 10.1097/MD.0000000000021849

**Published:** 2020-08-21

**Authors:** Yunbin Xiao, Wenfeng Li, Xicheng Deng, Zhi Chen, Yuming Peng, Yefeng Wang, Yunhong Zeng, Zhenghui Xiao

**Affiliations:** a Department of Cardiology, Hunan Children's Hospital; b Academy of Pediatrics, University of South China; c Department of General Surgery, Hunan Children's Hospital; d Intensive Care Unit, Hunan Children's Hospital, Changsha, Hunan, China.

**Keywords:** patent ductus venosus, pulmonary arterial hypertension, hypersplenism

## Abstract

**Introduction:**

Patent ductus venosus (PDV) is a rare and critical disease, and the majority of patients present with pulmonary arterial hypertension (PAH) or hepatopulmonary syndrome due to congenital portosystemic shunt. We reported that both PAH and hypersplenism were major complications of PDV in this case. This case report can assist the treatment and recovery of the patients with similar symptoms.

**Patient concerns:**

A 4-year-old male patient presented to our institution with a history of recurrent respiratory infections accompanied by leukocytopenia, thrombocytopenia and presented with tachypnoea. upon mild exertion.

**Diagnosis:**

A wide communication, 10 mm in diameter, between the portal vein and inferior vena cava was identified in the subcostal echocardiogram and computed tomography images. Echocardiography showed an estimated systolic pulmonary artery pressure of 106 mm Hg. Right-sided cardiac catheterization indicated a mean pulmonary arterial pressure of 30 mm Hg and a pulmonary vascular resistance of 3 Wood units. Chest X-ray revealed cardiomegaly with a prominent pulmonary segment.

**Interventions:**

The patient was treated with combination pharmacotherapy of bosentan and tadalafil and PDV ligation.

**Outcomes:**

A year later, the boy showed normal exercise tolerance and weight gain. Liver and spleen parameters, liver function, blood cells and the general condition of the boy improved.

**Conclusion:**

Initial combination therapy of bosentan and tadalafil is safe and effective in children with PAH associated with PDV. When PDV banding test shows normal portal pressure, PDV ligation is considered acceptable in children with PAH and hypersplenism associated with PDV.

## 1 Introduction

Patent ductus venosus (PDV), located between the portal vein and the inferior vena cava, is a rare congenital portosystemic shunt.^[1]^ The high-flow portal venous shunt has a certain effect on the liver, brain, and lungs, and there is a risk of developing liver dysfunction, portal shunt encephalopathy, and portopulmonary hypertension. However, there are a few reports on PDV with pulmonary arterial hypertension (PAH) and hypersplenism, and the appropriate management of this condition remains unknown. In particular, reports on ligation for the treatment of patients with PDV are scarce. Moreover, the indications, strategies, long-term efficacy, and safety of this therapy remain poorly understood. Here we report a child with PAH and hypersplenism caused by PDV; his PAH and hypersplenism were alleviated after ligation of the PDV.

## 2 Case presentation

**Figure 1:**
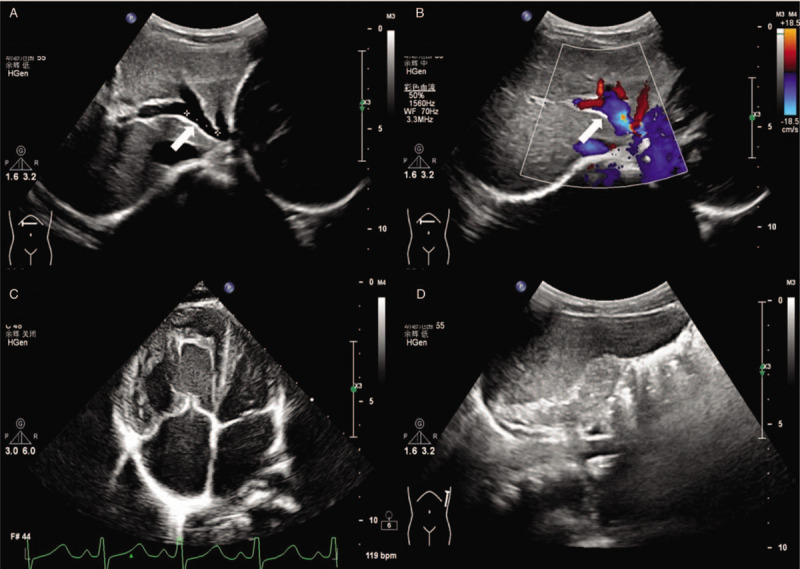
Abdominal ultrasound and echocardiogram. (A) the main branch of the portal vein and the patent ductus venosus can be seen, (B) the blood flow of patent ductus venosus can be seen, white arrow indicating ductus venosus, (C) echocardiography shows the enlargement of right ventricle and right atrium, (D) the enlargement of spleen can be seen.

**Figure 2:**
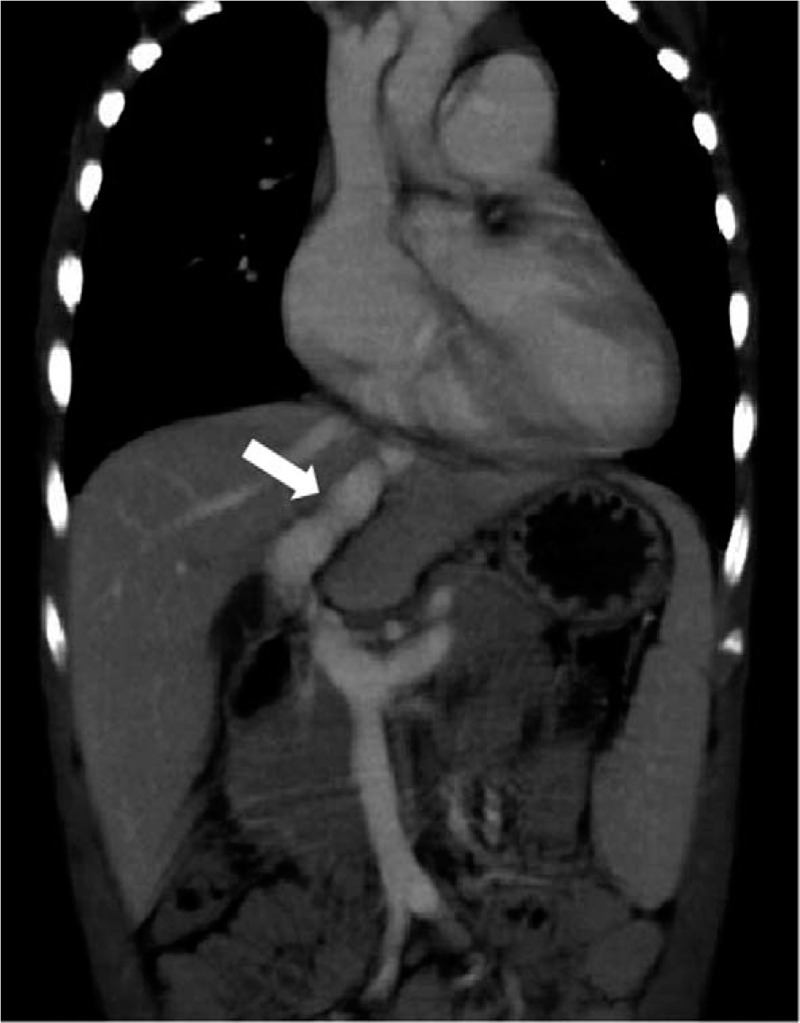
The main branch of the portal vein and the patent ductus venosus can be seen under computed tomography, white arrow indicating ductus venosus.

**Figure 3:**
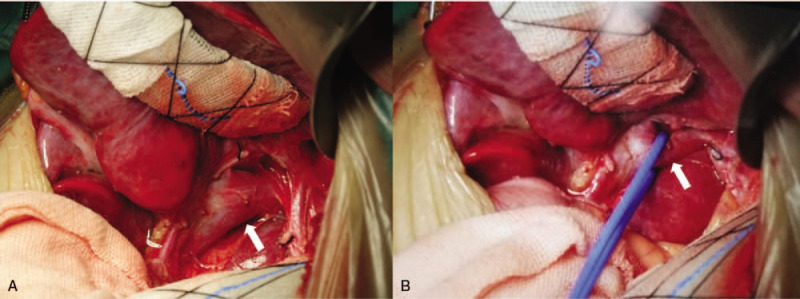
Surgical view of the PDV. (A) The liver was mobilized by a retractor. (B) The PDV was identified with a blue catheter, white arrow indicating ductus venosus. PDV = patent ductus venosus.

A 4-year-old male patient with a history of recurrent respiratory infections accompanied by leukocytopenia and thrombocytopenia presented with tachypnoea upon mild exertion. Physical examination revealed increased precordial activity, a heart rate of 110 bpm, an accentuated pulmonic component of the 2nd sound and a systolic murmur consistent with tricuspid regurgitation. The liver and spleen (Fig. 1) were enlarged. According to pulse oximetry, his blood oxygen saturation was 95%. NT-proBNP was 1008 pg/mL. His white blood cell count was 3.42 × 10^9^/L, platelet count was 62 × 10^9^/L, serum total bile acid was 54.3 μmol/L, serum aspartate aminotransaminase was 48.8 μ/L, serum total bilirubin was 23.6 μmol/L, serum direct bilirubin was 8 μmol/L, and the four index signs of blood coagulation were normal (Table 1). Chest X-ray revealed cardiomegaly with a prominent pulmonary segment. Echocardiography showed enlargement of the right ventricle, right atrium and pulmonary artery, with an estimated systolic pulmonary artery pressure of 106 mm Hg (Fig. 1), suggesting severe PAH. A wide communication 10 mm in diameter between the portal vein and inferior vena cava was identified in the subcostal echocardiogram (Fig. 1) and computed tomography (Fig. 2) images. To reduce pulmonary artery pressure, treatment with tadalafil (0.5 mg.kg^-1^ orally daily), bosentan (2 mg.kg^-1^ twice daily orally), furosemide (1 mg.kg^-1^ twice daily orally) and spironolactone (1 mg.kg^-1^ twice daily orally) was administered. One month later, the patient presented pancytopenia with a white blood cell count of 1.5 × 10^9^/L, platelet count of 19 × 10^9^/L, and red blood cell count of 2.79 × 10^12^/L, and a bone marrow smear indicated hypoplasia. Replacing furosemide with torsemide improved the pancytopenia and bone marrow hypoplasia. Six months later, his tachypnoea improved, and echocardiography showed the estimated systolic pulmonary artery pressure to be 58 mm Hg; NT-proBNP was normal. Right-sided cardiac catheterization was performed and low-dose heparin (50 U.kg^-1^) was administered, which resulted in a mean pulmonary arterial pressure (MPAP) of 30 mm Hg and a pulmonary vascular resistance of 3 Wood units. Before PDV ligation, a PDV banding test was performed. Immediately after PDV banding, the portal vein pressure increased from 23 mm Hg to 38 mm Hg and dropped to 21 mm Hg 15 minutes later; the MPAP was 25 mm Hg. PDV ligation was considered acceptable and subsequently performed (Fig. 3). The child tolerated the procedure well, and acute signs of portal hypertension did not occur. Half a month later, echocardiography showed that right ventricular internal dimensions decreased significantly. The clinical situation of the child improved. Blood platelets, blood ammonia, serum total bile acid, serum aspartate aminotransaminase, serum total bilirubin and serum direct bilirubin were all normal (Table 1). After PDV ligation, the wide communication between the portal vein and inferior vena cava could not be identified in the subcostal view of the echocardiogram (Fig. 1). One year later, the boy showed normal exercise tolerance and weight gain. Liver and spleen parameters, liver function, blood cells, and the general condition of the boy improved.

**Table 1:**
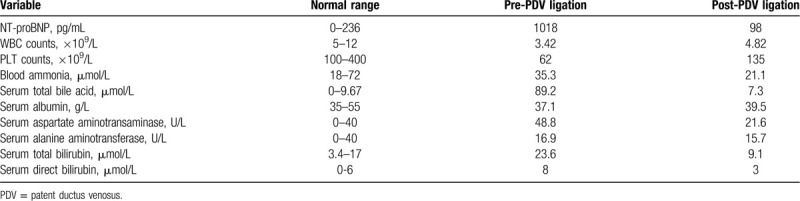
Laboratory data of the patient with PDV.

## 3 Discussion

We reported that PAH and hypersplenism were major complications of PDV in this case. Our patient developed severe PAH with increased pulmonary vascular resistance in the absence of organic liver disease, indicating that portosystemic shunt and reduced vasoconstrictors degradation may play important role in PAH development. Increased pulmonary artery pressure causes pressure elevation of the right heart, which might lead to hypersplenism via influencing inferior vena cava return and splenic congestion. Normal portal vein pressure after the PDV closure indicated that the PDV ligation was safe and effective.

In the fetal period, the umbilical vein and ductus venosus play critical roles in transporting oxygenated blood from the placenta to the heart. After birth, the ductus venosus normally closes spontaneously within 2 weeks.^[2]^ PDV is a rare form of congenital portosystemic venous shunt that can cause hepatopulmonary syndrome and PAH.^[3]^ PAH is a complication of PDV, portosystemic shunt, and long-term pulmonary vasoconstriction, thromboembolism, localized intimal fibrosis, and portal hypertension may contribute to PAH development.^[3]^ Large PDV in our patient induced significant portosystemic shunt and most part of pulmonary vasoconstrictor bypassed intrahepatic portal vein, which may contribute to development of PAH.

A 100% mortality rate was reported in patients who underwent liver transplantation when their MPAP was greater than 49 mm Hg.^[4]^ Since the patient was admitted to our center with severe PAH, the PDV closure procedure might have been fatal without a prior reduction in PAP. Bosentan, a dual endothelin receptor antagonist, lowers PAP and PVR and is well tolerated by children with idiopathic PAH.^[5]^ Tadalafil, a long-acting phosphodiesterase type 5 inhibitor, has good safety and potential efficacy in pediatric patients with PAH.^[6]^ With an initial combination therapy of bosentan and tadalafil, the exercise capacity in our patient improved in 6 months without elevated hepatic aminotransferase. Moreover, echocardiography showed that estimated systolic pulmonary artery pressure decreased from 106 mm Hg to 58 mm Hg. This result supports the safety and efficacy of bosentan combined with tadalafil therapy in children with PAH associated with PDV.

Therapeutic considerations in patients with PDV are inconsistent. Catheter embolization,^[2]^ stenting the PDV combined with iloprost administration^[3]^ and stepwise closure of the PDV^[7]^ have been reported. Stepwise surgical ligation may minimize the risk of developing acute portal hypertension at the expense of multiple procedures and possibly increase the combined risk to the patient. Transcatheter embolization could obviate the complex surgical ligation of PDV, but a one-step closure cannot prevent the risk of acute portal hypertension. Stent implantation may gradually close the PDV and spontaneously improve portal branch development. However, catheter procedures require intravenous high-dose heparin as an anticoagulant in both catheter embolization and stent implantation. Since our patient presented recurrent platelet count decreases associated with hypersplenism, to avoid heparin-induced thrombocytopenia resulting in aggravation, we decided to ligate the PDV with a surgical procedure. Portal pressure elevation post-PDV banding or balloon occlusion and hypoplastic portal branches indicate that the PDV should be closed gradually, not abruptly.^[3]^ Before PDV ligation, we performed the PDV banding test, which indicated that the portal pressure transiently increased after banding and recovered 15 minutes later in our patient. The PDV was ligated without clinical deterioration. We speculate that the portal branches were not hypoplastic in our patient, but the large PDV resulted in an insufficient opening of the portal branches by transferring most of the portal blood flow to the inferior vena cava; hence, the portal pressure spontaneously decreased when the portal branches gradually opened after PDV banding.

Hypersplenism is a disorder characterized by an enlarged spleen and the rapid destruction of blood cells.^[8]^ Hypersplenism is not common in patients with PDV. Our patient presented recurrent respiratory infections accompanied by leukocytopenia and thrombocytopenia and furosemide-induced bone marrow hypoplasia accompanying dramatic peripheral cytopenias. These conditions indicate that PDV can cause occult hypersplenism early, and infection or administered medicine can exacerbate hypersplenism by inducing bone marrow hypoplasia. Portal hypertension, caused by liver cirrhosis, is a major factor for hypersplenism.^[8]^ Our patient did not show symptoms of liver cirrhosis because of the extremely large PDV-connected portal vein and inferior vena cava. We speculate that the congestion of systemic circulation caused by severe PAH increased the portal vein pressure which leaded to splenic congestion. Moreover, the portosystemic shunt resulted in an insufficient opening or dysplasia of the intrahepatic portal veins, which may have worsened portal hypertension.

## Author contributions

**Conceptualization:** Yunbin Xiao, Zhenghui Xiao.

**Data curation:** Wenfeng Li, Yuming Peng, Yunhong Zeng.

**Formal analysis:** Zhenghui Xiao.

**Investigation:** Zhi Chen, Yefeng Wang.

**Resources:** Yunbin Xiao, Zhenghui Xiao.

**Supervision:** Zhenghui Xiao.

**Validation:** Yunbin Xiao, Zhenghui Xiao.

**Visualization:** Zhi Chen, Yefeng Wang, Yuming Peng.

**Writing – original draft:** Yunbin Xiao, Wenfeng Li.

**Writing – review & editing:** Zhenghui Xiao, Xicheng Deng, Yunbin Xiao.
